# Evidence centered design framework and dynamic bayesian network for modeling learning progression in online assessment system

**DOI:** 10.3389/fpsyg.2022.742956

**Published:** 2022-07-22

**Authors:** Younyoung Choi, Robert J. Mislevy

**Affiliations:** ^1^Department of Psychology, Ajou University, Suwon, South Korea; ^2^Department of Human Development and Quantitative Methodology, University of Maryland at College Park, College Park, MD, United States

**Keywords:** dynamic bayesian networks, evidence-centered design, assessment, learning progressions, learning analytics

## Abstract

An overarching mission of the educational assessment community today is strengthening the connection between assessment and learning. To support this effort, researchers draw variously on developments across technology, analytic methods, assessment design frameworks, research in learning domains, and cognitive, social, and situated psychology. The study lays out the connection among three such developments, namely learning progressions, evidence-centered assessment design (ECD), and dynamic Bayesian modeling for measuring students’ advancement along learning progression in a substantive domain. Their conjunction can be applied in both formative and summative assessment uses. In addition, this study conducted an application study in domain of beginning computer network engineering for illustrating the ideas with data drawn from the Cisco Networking Academy’s online assessment system.

## Introduction

An overarching mission of the educational assessment community today is strengthening the connection between assessment and learning (Oliveri and Mislevy, 2019; Gordon, 2020). To support this effort, researchers draw variously on developments across technology, analytic methods, assessment design frameworks, research in learning domains, and cognitive, social, and situated psychology. Coordinating insights from such disparate areas, each pushing the boundaries of familiar assessment practices, presents its own challenges.

Likewise, while substantive, psychological, instructional, and task developmental aspects of learning progression have been considered, few assessment design frameworks have been proposed to link the theory embodied in a learning progression, tasks that provide evidence about a student’s level on that progression, and psychometric models that can link them. More specifically, few statistical analytic models have been studied to characterize the relationship between student performance and levels on learning progressions.

This paper describes and illustrates the connections of three core components which are an evidence-centered design (ECD) framework for designing assessments (Mislevy et al., 2003b), learning progressions (LPs; Alonzo and Gotwals, 2012) that describe research-based paths of content and skills in a learning area, and a measurement model called dynamic Bayesian networks (DBNs, Murphy, 2002). The coherence of three core components above supports the development and use of assessments integrated with instruction, to provide information to guide students’ learning in formative and summative uses. This study also presents an application study using data from the domain of beginning computer network engineering, drawn from a renovation of curriculum, instruction, and assessment in the Cisco Networking Academy (CNA). In the application study, dynamic Bayesian networks was conducted with data from the CNA online assessment system, specifically the four-semester Cisco Certified Network Associate (CCNA) course sequence for assessing a learner’s status and change in connection with instruction based on an LPs.

## Learning progression, assessment, and Bayesian network

### Connecting learning and assessment

Black and Wiliam (1998) argue that to best support learning, an assessment must produce evidence of a student’s level of knowledge, skills, and abilities (KSAs) and elicit performance associated with demonstrating the state of KSAs expected at that level. They suggest a combination of cognitive theory of learning, assessment design, measurement models, and curriculum provides the most beneficial information to promote student learning. Expressing a similar viewpoint for assessment more generally, the National Research Council (2001, 2014) proposed an Assessment Triangle that emphasizes the theoretical and empirical connections among cognitive/substantive theory, task design, and analytic methods in order to create valid assessment and support reliable inferences for the purpose at hand, whether formative, summative, certification, etc. Therefore, a central challenge in assessment design is developing suitable frameworks that link theory, tasks that provide observable evidence about a student’s capability relative to those substantive theories, and analytic models that interpret student performance accordingly.

Formative assessment in particular is of increasing interest, where the objective is assessing students’ learning progress during instruction in order to guide further learning, rather than focusing on achievements at their end of a program of study (Black and Wiliam, 1998; Elwood, 2006; Bennett, 2011; Briggs et al., 2012). Huff and Goodman (2007) found that a large percentage of teachers wished they had more individualized diagnostic information from these assessments. The National Research Council (2001, 2014) reported that formative and timely feedback is important to students in their learning development (also see Gordon, 2020). One of the major purposes of formative assessment is to check student’s progress on learning tasks as well as to monitor the effectiveness of the teachers’ instruction, which can result in identifying a gap between a student’s actual level and desired level of performance, thus providing information to bolster a student’s understanding of a topic (Havnes et al., 2012; Kusairi et al., 2019).

Further, the COVID-19 pandemic has challenged education institutions around the world in teaching by traditional means. This crisis prompts administrators to adopt alternative strategies to address learning assessment (Khan and Jawaid, 2020). Online formative assessments are increasingly of interest as an alternative solution, which can be implemented by mailing/e-mailing, messaging platforms, discussion boards, and online educational platform tools (Nagandla et al., 2018; Choi and McClenen, 2020; Khan and Jawaid, 2020). For examples, CAN provides online educational platform in high schools, colleges, and community organizations around the world, drawing on curricula, instruction, and interactive assessment.

An evidence-centered design framework (ECD; Mislevy et al., 2003b) is a principled assessment design framework that can provides guidance for generating tasks that evoke evidence about students’ KSAs, and for coherently connecting theory embodied in an application with task design, and for choosing analytic models that best characterize the relationship between them. Furthermore, Arieli-Attali et al. (2019) proposed an expanded ECD including a learning layer as assessment design framework. The expanded ECD supports a creation of a system for blended assessment and learning at the design stage. The learning layer consists of (1) e-Proficiency model for identifying learning processes, (2) e-Task model for specifying features of learning support in the task design, and (3) e-Evidence model for addressing statistical methods of inferring latent learning processes. In a similar vein, Deonovic et al. (2020) proposed a master model that incorporates learning and assessment. The master model provides latent-variable statistical model that supports detailed diagnostic feedback related to the learning model.

### Learning progressions

Learning progressions provide a grounded theory for creating tasks and making inferences about a student’s progress. Interest in LPs has been increasing in many educational areas because they provide substantive evidence in the development of formative assessment (Arieli-Attali et al., 2019). LPs are defined by measurable pathways that a student may follow in the process of developing their knowledge and gaining expertise over time (National Research Council, 2001). Wilson’s (2012) research on measurement for structured assessments focused on learning progressions defined by means of construct maps.

Generally, an LP consists of several ordered levels or units, each of which represents a given state of KSAs required for a student to achieve mastery at that level. Major objectives in the study of an LP are to provide (1) information regarding the state of a student with respect to the level of understanding of a given concept and (2) diagnostic information regarding the strength and weakness of a student’s understanding along a curriculum (Gotwals et al., 2009; Schwarz et al., 2009; Briggs and Alonzo, 2012; West et al., 2012). To provide such information about the learning states of a student (i.e., the current, past, and prospective future levels of a student on LPs), the first step is to develop tasks for gathering student responses that provide evidence about students’ KSAs in relation to their levels on LPs. More specifically, if key task features that can evoke evidence about student states have been identified by drawing on research, then the information can be used for constructing tasks that can elicit student responses containing evidence about student KSAs. Through the assessment design framework, tasks are generated to reflect the targeted aspects of KSAs by incorporating the identified task features that evoke evidence about the KSAs or the targeted strategies. As an exemplar of theory-based task design, Embretson’s (1998) “cognitive design system” integrates the principles of cognitive psychology into task design.

In a study of LPs, Gotwals et al. (2009) presented a set of designed tasks linked to the LP of inquiry reasoning to gather evidence of how students use their content knowledge to formulate scientific explanations associated with a range of ecology, classification, and biodiversity domains. As an example, Table 1 shows a scenario for assessing the concept of biodiversity. Given the scenario, two tasks are generated relative to different levels of the LP of biodiversity. Both tasks ask the student to provide an answer and a rationale for their answer. Question A, relating to a lower level of the LP, asks students to identify which zone has the highest animal richness. The answer and its evidence are straightforward in the task because Zone B clearly has the highest animal richness. In contrast, Question B, related to a higher level, asks students to identify which zone has the highest biodiversity, given the same scenario. While the answer is the same as the previous task, providing appropriate and sufficient evidence supporting the answer is not as straightforward because students need to understand the difference between the concepts of the *richness* and the *abundance* of animals. Therefore, by using the key task features associated with the LP, a teacher obtains evidence about the level a student may have attained and what a student knows with respect to the LP domain.

**TABLE 1:** An example of a task taken from Gotwals et al. (2009).

School Yard Animal Data			
Animal Name	Zone A	Zone B	Zone C
Pillbugs	1	3	4
Ants	4	6	10
Robins	0	2	0
Squirrels	0	2	2
Pigeons	1	1	0

**Question A**		**Question B**	

Which zone has the highest richness, given this scenario?		Which zone has the highest biodiversity, given this scenario?	

Scenario 1: this table shows school yard animal data collected using CyberTracker. Use the table to help you answer the question.

Once tasks have been developed, another major issue is modeling the relation that links student performance on assessment tasks to their levels on the LP. Historically, measurement of proficiency change in accordance with development theory, cognitive psychology, and learning science has been a significant issue in educational and psychological research, such as Piaget’s (1950) stages of cognitive development, Siegler and Campbell’s (1989) multiple strategies in proportional reasoning ability of children, and Rock and Pollack-Ohls’s (1987) math learning as a dynamic latent variable consisting of a series of discrete stages. Various approaches in psychometric models have been proposed for addressing the measurement of proficiency change.

### Psychometric models for learning progressions

Historically, measurement of proficiency change in accordance with development theory, cognitive psychology, and learning science has been a significant issue in educational and psychological research, such as Piaget’s (1950) stages of cognitive development, Siegler and Campbell’s (1989) multiple strategies in proportional reasoning ability of children, and Rock and Pollack-Ohls’s (1987) math learning as a dynamic latent variable consisting of a series of discrete stages. Various approaches in psychometric models have been proposed for addressing the measurement of proficiency change. A suitable psychometric model requires certain properties for addressing learning progressions: (1) Observations involve student performances in task situations; most often, observables are categorical variables such as from selected response items or human or automated evaluation of more open performances; (2) a learning progression is operationalized as a latent variable with several ordered latent classes representing qualitatively different levels in the learning progression; and (3) there is a structural, generally probabilistic, latent-variable relation in an LP over time when the variable of interest is unobservable. (4) Task design and theory provide a theoretical framework for creating and modeling observable evidence as well as information about the nature and structure of expected change.

Some psychometric models matched to LP research have been proposed in Latent Class Analysis (McCutcheon, 1987), Rule Space model (Tatsuoka, 1990), Cognitive Diagnosis models (CDM, Leighton and Gierl, 2007), Mixture IRT (Sen and Cohen, 2019), Bayesian networks (Madigan et al., 1995), and hidden Markov models (Wiggins, 1955; Collins and Wugalter, 1992). Most research in CDMs and rule space modeling has focused on the classification at a given time point; the movements from one attribute at one point in time to others at the next point in time, as might be expressed in terms of transition proportions of skills, levels, and strategies between consecutive measurement time points, are not addressed.

Markov chain models can describe transition proportions of latent classes between consecutive time points. These models have been applied in situations such as attitude change, learning, cognitive development, and epidemiology (Langeheine and Van de Pol, 2002). Variations of Markov chain models (i.e., hidden Markov models, mixed hidden Markov models, and mixed hidden Markov models with multiple groups) have been proposed (Langeheine and Van de Pol, 2002). The models concern modeling change over time in observed categorical variables by using transition probabilities for unobserved variables. The hidden Markov model thus combines features of a latent class model and those of a simple Markov chain model. The model is also referred to as a latent Markov model, as proposed by Wiggins (1955) or a Latent Transition Analysis (Collins and Wugalter, 1992). This model has been applied to identify unobservable latent state changes such as strategies, levels, and skills based on observable student responses at each point in time.

The conditional probabilities and transition probabilities of a hidden Markov model correspond to the expression of a dynamic Bayesian network, or DBN (see Dean and Kanazawa, 1989, for an introduction to DBNs and Reichenberg, 2018, for a recent review of their use in educational assessment).

Dynamic Bayesian networks are an extended model of Bayesian Networks, which are a probability-based flexible statistical modeling framework rather than a specific statistical model. This framework supports reasoning and decision-making with uncertain and inconsistent evidence. BNs use graphical representation and linked probability theory (Kjaerulff and Madsen, 2007). The graph consists of nodes representing unobservable and observable variables and directed edges representing stochastic or logical relations among variables. The joint probability distribution of a set of (typically finite-valued) variables is represented recursively as the product of conditional distributions,


$$P\left(A_{i}=a_{i}|pa\left(A_{i}\right)\right)$$

where the “parents” of a node *A* are the nodes with edges from them to *A*. If *A*_*i*_ has no parents, the conditional probability is regarded as a marginal probability. An ordering of the variables for the recursive expression is selected to take advantage of conditional independence relation. In particular, latent variables are typically modeled as parents of observable variables. The formal expression of the joint distribution is


$$P\left(A_{i}=a_{i},\ldots ,A_{n}=a_{n}\right)=\prod P\left(A_{i}=a_{i}|pa\left(A_{i}\right)\right)$$

A conditional probability table (CPT) for *A*_*i*_ has columns for each of its possible values, and rows for each possible combination of values of its parents pa(*A*_*i*_) that are conditional probability distributions for *A*_*i*_ conditional on the given values for pa(*A*_*i*_).

In assessment applications, evidence is received from external sources in the form of observable variables such as task responses or raters’ scores. A likelihood distribution over the states of its parent variables, typically but not necessarily latent variables representing KSAs, is induced by a value of an observable variable. Once all of interrelationships are expressed in terms of the recursive representation of the joint distribution of variables, it is possible to calculate the updated states of any variables effected by new information about another set of variables via Bayes’ rule. As the size of the collection of variables and the complexity of their interrelations increases, computational algorithms more efficient than definitional application of Bayes rule can be applied (e.g., Lauritzen and Spiegelhalter, 1988).

## Illustration

### Dynamic Bayesian networks

The preceding sections describe how LPs and ECD can provide a coherent, theory-based assessment design framework in formative assessment, and how Bayesian networks can be used to manage issues of evidence and inference. This section describes how DBNs can be used to model learning progressions over multiple time points in such a system. Specifically, it addresses the questions of how the current, past, and future levels of a student’s LPs are related and can be inferred from student responses. We note that DBNs are an extension of the hidden Markov model to represent multivariate latent and discrete spaces. Taken together, the discussions explain how the DBNs can model LPs over time by connecting defined LPs, assessment design, and the interpretation of student performances.

In this study, DBNs can be used to model learning progressions over multiple time points under a formative assessment system. DBNs have been applied to intelligent tutoring systems for modeling changes in students’ knowledge. As examples, Reye (1996, 1998) used dynamic belief networks in the intelligent tutoring system. Chang et al. (2006) developed a Bayes net toolkit for student modeling in an intelligent tutoring system. Almond (2007, 2010) extended ECD to incorporate DBNs, in terms of a partially observable Markov decision process (POMDP). As an example, Almond used the POMDP to model student growth in a study of simulated music tutoring program. DBNs thus offer a psychometric model to link the theory embodied in a learning progression and tasks that provide evidence about a student’s level on that progression through ECD assessment design framework. That is, DBNs can support inference about students’ levels and expected change in an LP over time when the variable of interest is unobservable, when task design and theory provide a theoretical framework for creating and modeling observable evidence as well as information about nature and structure of expected change.

Dynamic Bayesian networks are an extension of hidden Markov models (HMM) that is used to represent multivariate latent and discrete spaces. The elements and structural relationships in DBNs correspond to a standard algebraic expression of HMMs through the concept of Markov property and conditional independence. An HMM is comprised of a Markov chain and observables (Cappé et al., 2005). A Markov chain is a sequence of discrete random variables with the Markov property. The term “hidden” refers to the fact that variables of the Markov chain are latent; that is, their values are never directly observed, although evidence about them arrives in the form of observable variables that depend on them stochastically. Three kinds of distribution parameters are then to be estimated: (1) the initial multinomial state distribution, P(X_*t=*1_) in a DBN; (2) the transition model, set of conditional multinomial distributions that represent the transition probabilities P(X_*t*_ | X_*t–*1)_; and (3) the observation model, which corresponds to the conditional probability distributions of observables Y_*t*_, or P(Y_*t*_ | X_*t*_) in the DBN.^1^ The formal probabilistic notation of the hidden Markov chain in the HMM is denoted as follows:


$$P\,\left(X_{t+1},\ldots ,X_{1}\right)=P\,\left(X_{t+1}|X_{t}\right)\times \ldots \times P\,\left(X_{2}|X_{1}\right)\,P\,\left(X_{1}\right)=\prod P\,\left(X_{t}|X_{t-1}\right)$$

with *P*(*X*_1_|*X*_0_) interpreted as *P*(*X*_1_). Under the assumptions of conditional independence and the first-order Markov property, the observations {*Y*_*t*_} are independent given the states of a hidden Markov chain {X_*n*_} at all time points but *X*_*t*_:


$$P\,\left(Y_{1},\ldots ,Y_{n}|X_{1},\ldots ,X_{n}\right)=\sum_{t}P\,\left(Y_{t}|X_{t}\right)$$

Dynamic Bayesian Networks thus extend static Bayesian Networks (BNs) to model probability distributions over multiple time points (Murphy, 2002). A common approach to representing DBNs is to combine multiple static BNs for a desired number of time slices (Kjaerulff and Madsen, 2007). Therefore, the DBNs can be used for inference about previous states, current states, and possible future states of a system over time (Murphy, 2002). A DBN contains a prior for the initial hidden state, P(X_1_), a transition function of the hidden states over multiple time points, P(X_*t*_ | X_1:t–1_), and observable variable(s) given each hidden state, P(Y_*t*_ | X_*t*_).

This discussion assumes three properties. First, the links of time slices are defined by the conditional probability of the variables at a current Time t given the variables at previous Time t-1, the first order Markov property. Second, observations are structured under assumptions of conditional independence, in that P(Y_*t*_) is conditionally independent of P(Y_*t‘*_), given X_*t*_ for t ≠ t‘. Third, the sense of “dynamic” refers to state change over time, not network or structure change over time. Under the three assumptions, the formal notation of DBN at Time t can be expressed with respect to a graphical model (Murphy, 2002) as


$$P\,\left(A_{t}|A_{t-1}\right)=\prod P\,\left(A_{t}^{i}|p\,a\,\left(A_{t}^{i}\right)\right)$$

where A_*t*_ = (X_*t*_, Y_*t*_), incorporating the latent variable (X) and the observation (Y) at time t and $A_{t}^{i}$ is the *i^th^* element of this concatenation. The joint probability distribution is then as follows:


$$P\,\left(A_{1:t}\right)=\prod_{t=1}^{T}\prod_{i=1}^{N}P\,\left(A_{t}^{i}|p\,a\,\left(A_{t}^{i}\right)\right)$$

Again it is possible to calculate the updated states of any variables as affected by new information about another set of variables through Bayes theorem, directly or by more efficient algorithms.

In Bayesian networks with discrete variables, Bernoulli and categorical distributions are used for the conditional probability distributions. Their parameters are incorporated into the full model and can also be learned from the observations, using Beta and Dirichlet distributions as conjugate priors for them (see Almond et al., 2015, Chap. 8, for more parsimonious prior distributions that can be employed when substantive theory is available). Bayesian inference is based on posterior distributions of variables of interest, obtained by combining the prior distribution on the variable(s) of interest with the likelihood induced by observations through the appropriate conditional distributions. A common point estimate is the maximum *a posteriori* (MAP) estimate; that is, the value that yields the maximum posterior probability for an unobserved variable given the realized data. In LP research, the values representing the levels of LPs are not directly observed. The MAPs for an LP variable are the levels that are most probable for each student given their responses.

Expectation and Maximization (EM) algorithms, gradient ascent, and Markov chain Monte Carlo Estimation (MCMC) are commonly used in BN software programs to estimate the values of the parameters of the distributions of the latent variables. This study uses the EM algorithm (Dempster et al., 1977) as implemented in Netica (Norsys Software Corp, 2010) to estimate parameters of DBNs.

### Dynamic Bayesian networks with learning progressions

**FIGURE 1:**
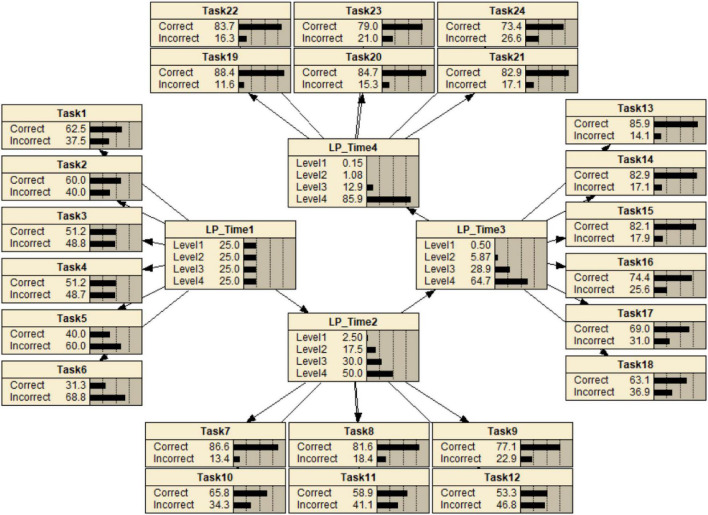
An initial representation of DBN for modeling LPs.

As an illustration of DBNs, we suppose that the same students are repeatedly measured at more than one point during a period of instruction (e.g., a course in a semester or an intervention). The tasks used are designed by incorporating task features with which students can be differentiated in terms of levels of understanding or achievement that are theoretically grounded in a substantively based learning progression theory. In such situations, the investigation of the patterns of change in student levels across measurement occasions can offer diagnostic information customized to reflect individual learning and provide an informative evaluation of the effectiveness of instruction. For a simple example of modeling LPs through the DBNs, suppose that four measurements are designed. At each time point, there is a latent variable representing an LP and the observables that depend on them in probability. It is assumed that three levels are identified in the LP. Each measurement consists of six tasks across time points. Figure 1 shows an example of modeling LPs with a DBN.

In this example, the probabilities of the initial marginal probability table of the first measurement, CPTs, and transition probability tables are hypothetically set in order to illustrate the structure of DBNs. They could be estimated from observations or determined through theory or domain expert opinions. In this example, the hypothesized transition probabilities are restricted in such a way that all the probabilities of reverse changes are zero. This constraint reflects an LP considering only forward movements over time. Other types of transition probability patterns can be considered depending on different substantive theory such as a forward movement, an adjacent movement, and all possible movements. The different patterns can be modeled by (1) constraining sets of transition probabilities to be equal to zero, (2) restricting them to be a particular value, or (3) fixing them to be equal to each other such as a forward movement, an adjacent movement, and all possible movements.

**FIGURE 2:**
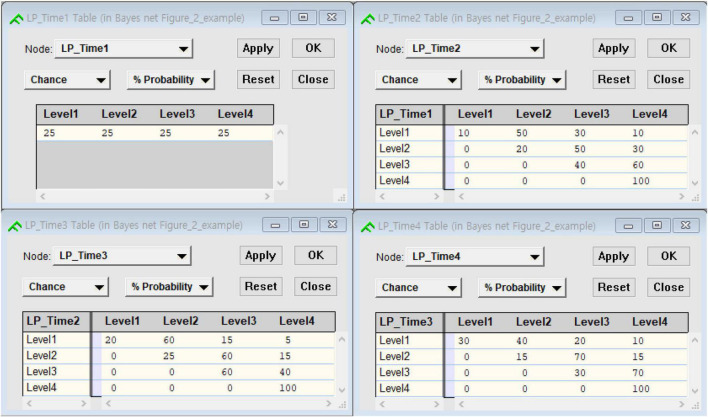
Initial probability matrix and transition probability matrices.

**FIGURE 3:**
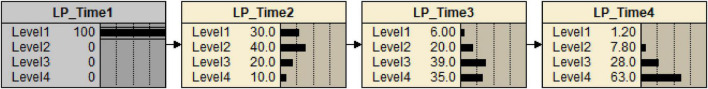
A DBN representation of the four latent variables without tasks.

To understand how the transition function works for the purpose of investigating state change over time, one could consider a situation where student status at the first measurement occasion is known. This information is propagated through the network by Bayes theorem. The posterior distribution of the next three variables given the student’s states at the first measurement occasion can be updated by using the transition function (see Figure 2, which shows an initial probability table at the first measurement occasion and transition probability tables). Figure 3 shows the posterior distributions of three variables (i.e., LP_Time2, LP_Time3, and LP_Time 4) given a student latent Level 1 at the first measurement occasion. The grayed-out coloring of the first node indicates that the value of that variable is known with certainty at the point in time at issue. It can be inferred that the student is most likely at Level 2 at the second measurement occasion with 0.40, at Level 3 with 0.39 at the third measurement occasion, and at Level 4 with 0.63 at the fourth measurement occasion, respectively.

**FIGURE 4:**
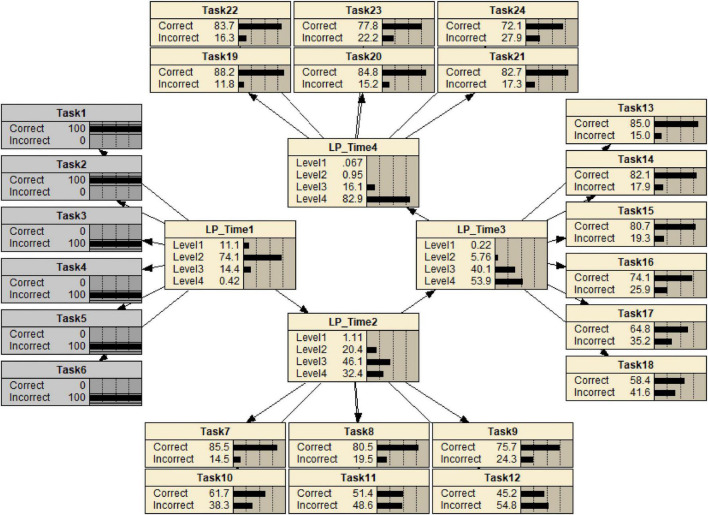
A representation of the DBN when the student has the particular response pattern given 6 tasks at the first measurement occasion.

The next step considers the DBNs with tasks. The conditional probability distribution for Task 1 given the first measurement occasion is shown in Table 2 as an example; note that this is a task that is most useful for differentiating students at Level 1 from Levels 2–4. Once a student’s responses have been observed at any given time point, this information is propagated through the network. The posterior distributions of the latent variable at that time point as well as the latent variables at previous and future time points are obtained. Figure 4 shows a situation in which observations are [1,1,0,0,0,0], indicating that Tasks 1 and 2 are correct while Tasks 3, 4, 5, and 6 are incorrect at the first measurement occasion.

**TABLE 2:** Conditional probability table for Task 1 given Learning progression at the first measurement occasion (% Probability).

		Correct	Incorrect
LP_ Time 1	Level 1	10	90
	Level 2	70	30
	Level 3	80	20
	Level 4	90	10

Based on these observations, the posterior distributions of the four latent variables are updated. Note that the first six Task variables are shaded, indicating that their values are known, while the node for LP_Time1 is not shaded because knowledge about its value is improved by the information in the responses but is still not known with certainty. This shows that the student is more likely to be at Level 2 at the first occasion with 0.74, at Level 3 at the second with 0.46, at Level 4 at the third with 0.54, and at Level 4 at the fourth with 0.83 when the student has the particular pattern of responses to the six tasks at the first measurement occasion.

## Application

### Data

An application study was conducted with data from a blended online-classroom course provided by the CNA. Each course contains several chapter exams and a final exam. The same students were measured several times over the course of the curriculum. The target populations of the courses are high school students, 2- and 3-year community college and technical school students, and 4-year college and university students. The study addressed a learning progression concerning IP (Internet protocol) addressing. The 26 tasks are modeled as conditionally independent observable variables on a single discrete latent variable with values that indicate four levels of the LP. The student sample size was 1,450. A DBN was constructed and observations collected on two occasions approximately one month apart.

### Learning progression for internet protocol addressing skill

The learning progression for IP Addressing skills was identified as a subset of larger pool of assessment items, based on a combination of confirmatory discrete factor analyses and subject matter experts’ opinion (as further described in West et al., 2010, 2012). The analyses were conducted using M*plus* (Muthén and Muthén, 2006) and the fit of competing models was carried out with the BIC and the bootstrapped likelihood ratio test (McLachlan and Peel, 2000; Nylund et al., 2007). Table 3 shows the four levels in the resulting LP. Each level contains the descriptions of KSAs, where Level 1 can be defined as describing novice students that possibly have pre-course KSAs.

**TABLE 3:** A set of levels of IP Addressing Skill.

Level 1 – Novice -	1 2 3 4 5 6	Student can navigate the operating system to get to the appropriate screen to configure the address. Student knows that four things need to be configured: IP address, subnet mask, default gateway and DNS server. Student can enter and save IP addressing information that has been provided. Student can use a web browser to verify network and or Internet. Student can verify that the provided information was correctly entered. Student knows that DNS translates names to IP addresses.

A learner knows pre-course knowledge and skills in IP addressing skills		

**Level 2** – **Basic** –	1	Student understands that an IP address corresponds to a source or destination host on the network.
	2	Student understands that an IP address has two parts, one indicating the individual unique host and one indicating the network that the host resides on.
	3	Student understands how the subnet mask indicates the network and host portions of the address.
	4	Student understands the concept of local –vs.- remote networks.
	5	Student understands the purpose of a default gateway and why it must be specified.
	6	Student knows that IP address information can be assigned dynamically.
	7	Student is able to create a simple IP addressing scheme based on host or network requirements.
	8	Student can describe the need and features of IPv6 addresses.

A leaner knows fundamental concept of IP addressing		

**Level 3** **– intermediate –**	1	Student understands the difference between physical and logical connectivity.
	2	Student understands the difference between Layer 2 and Layer 3 networks.
	3	Student understands that a local IP network corresponds to a local IP broadcast domain. (both the terms and the functionality)
	4	Student knows how a device uses the subnet mask to determine which addresses are on the local Layer 3 broadcast domain and which addresses are not.
	5	Student can use the subnet mask to create an addressing scheme that accommodates design requirements for number of hosts per subnet and number of networks.
	6	Student understands why the default gateway IP address must be on the same local broadcast domain as the host.
	7	Student understands the ARP process and the role of Layer 2 addresses within a Layer 3 broadcast domain.
	8	Student knows how to interpret a network diagram in order to determine the local and remote networks.
	9	Student understands how DHCP dynamically assigns IP addresses.
	10	Student knows the purpose of private, public, and special reserved addresses such as multicast and loopback, IP address spaces and when to use either one.
	11	Student recognizes reserved IPv6 addresses.

A leaner knows more advanced concepts of IP addressing		

**Level 4** - **Advanced**	1	Student can create an IP addressing scheme for a network using VLSM
	2	Student can use a network diagram to find the local network where the configured host is located.
	3	Student can use a network diagram to find the other networks attached to the local gateway device.
	4	Student can use the PING utility to test connectivity to the gateway and to remote devices.
	5	Student can recognize the symptoms that occur when the IP address or subnet mask is incorrect.
	6	Student can recognize the symptoms that occur if an incorrect default gateway is configured.
	7	Student can recognize the symptoms that occur if an incorrect DNS server (or no DNS server) is specified.
	8	Student knows why DNS affects the operation of other applications and protocols, like email or file sharing.
	9	Student can use NSlookup output to determine if DNS is functioning
	10	Student can create a DHCP addressing scheme recognizing the importance of excluding addresses.
	11	Student is able to convert an IPv4 address to an IPv6 address.

A leaner can apply knowledge and skills in context of IP addressing		

### Evidence-centered design framework for modeling internet protocol addressing skill

The Conceptual Assessment Framework in the ECD framework provides technical specifications for the evidentiary arguments that the operational assessment embodies. Modeling the LPs for IP Addressing is organized around five guiding questions (see Mislevy et al., 2003a,b, for further details in the general case, and Zalles et al., 2010, for the case of learning progressions):

(1) Construct Model: What complex of knowledge, skills, or other attributes should be assessed?

**FIGURE 5:**
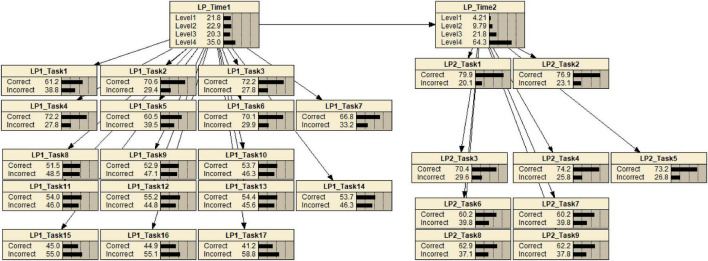
A DBN for an LP for IP addressing skills, before observations.

The construct model variables can be specified by the aspects of KSAs associated with levels, progress, levels of learning progressions, and diagnostic information. A construct model variable can represent either an LP of a particular domain when the levels are ordered, or as a level of a particular LP when the ordering may be partial. The construct model shows that the levels are defined for assessing the LP in the domain and information regarding the KSAs that are required for students at that level. In addition to this information, the structure of the construct model variables is also specified. For this LP, based on expert opinion and the preliminary analyses, a hierarchical relationship among the construct model variables is posited to be an adequate structure. We used two instances of the same four-level IP Addressing LP variable for the two occasions. The LP variable at the first measurement time point influences the second LP variable at the second time point. This construction becomes the representation of latent variables in a DBN (Figure 5). The construct model is connected with the task model through the evidence model, which explains how each observable depends on the construct model variables.

(2) Evidence Model: What behaviors or performances should reveal those constructs? How are they connected?

For the study of LPs, the evidence model provides (1) information about how student performances are modeled and interpreted relative to the level of an LP, (2) information about the criterion for comparing observed and expected LPs, and (3) information about feedback within and across task level. Consequently, the evidence model provides inferential reasoning from observables of tasks and expectations in the student model. This study illustrates these relationships with a DBN, one of the suitable psychometric models for modeling LPs. The DBN of this LP model after estimating the CPTs from the data set of 26 items and 1450 students is shown in Figure 5 in the results section. Task model: What tasks or situations should elicit these behaviors?

**FIGURE 6:**
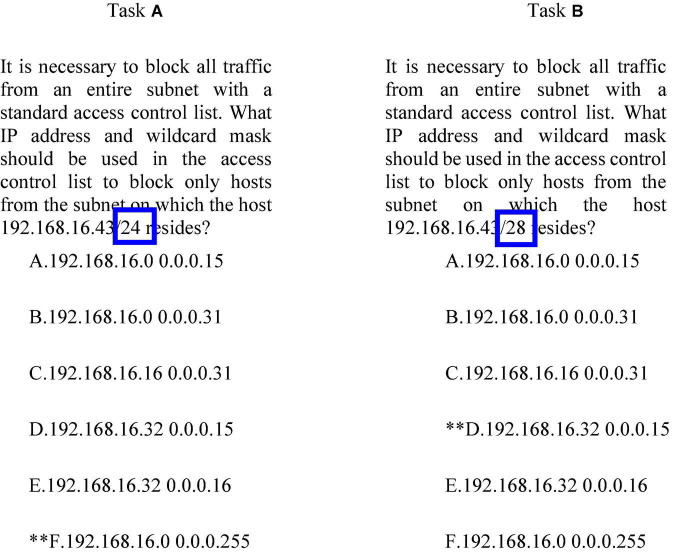
An example of a task taken from West et al. (2012).

For assessing LPs, the task model provides information for developing tasks to elicit student performances relative to the levels of a learning progression. Specifically, it contains the following information: (a) the key features of tasks that are important to elicit student’s understanding with respect to the targeted KSAs at a particular level of an LP, (b) the key features of tasks, which are more likely to classify student performances into different levels of an LP, (c) the key features that make a task more or less difficult, (d) other characteristics/contexts of a task that affect its difficulty, and (e) the aspects and features that inform the quality of tasks for assessing LPs. By designing assessment tasks that target different levels associated with different aspects of targeted KSAs, it becomes possible (a) to infer the level of the LP a student may have attained, (b) to draw conclusions about the value, sequence, and structure of a student’s learning, and (c) to gather empirical evidence to guide the development and refinement of the hypothesized LPs associated with assessment and curriculum. West et al. (2010, 2012) identified the features of tasks relative to different levels of the LP in IP Addressing Skills. As an example, two tasks in Figure 6 require different levels of IP Addressing skills to obtain the correct answer. The two tasks look similar on the surface, but the stem of Task A is/24, while that of Task B is/28. This change requires students to use a more advanced IP addressing skill (specifically, reasoning across octal boundaries; West et al., 2010, 2012).

Appropriate and sufficient evidence with respect to levels in LPs can be obtained by incorporating task features associated with the key aspects of knowledge and skill required of students to complete the tasks. This study used tasks identified by their features relative to levels of the IP Addressing Skills progression. LP1_Task1 to LP1_Task7 and LP2_Task1 to LP2_Task3 are the tasks identified by the features relative to Level 2 of the IP Addressing Skills progression. LP1_Task 8 to LP1 Task 14 and LP2 Task 3 to LP2_Task 5 are the tasks identified by the features relative to Level 3 of the progression. Lastly, LP1_Task15 to LP1_Task17 and LP2_Task6 to LP2_Task9 are the tasks identified by the features for assessing Level 4.

(3) Assembly model: How much do we need to measure?

For assessing LPs, the assembly model describes how the three models above are combined for inferring a student’s level on a learning progression in a given assessment situation. For instance, the number of tasks (i.e., test length) with respect to the different levels on an LP and the balances of task type and focal KSAs are determined to construct an optimal assessment.

(4) Presentation model: How does the assessment look?

The presentation model describes how a task is presented to students. There are many different means for delivering an assessment, such as paper and pencil format, computer and web-based format, and simulation- and game-based format. The requirements for presenting assessments differ depending on the format. Formative assessment can also be delivered using mailing/e-mailing, messaging platforms such as Messenger and WhatsApp, and online educational platform tools, such as Questbase and Woot Math. The presentation model also contains specifications for the interactions between the student and the assessment, and the work products that will be captured for subsequent evidence identification.

### Results

#### Dynamic Bayesian networks

Figure 5, introduced previously, shows the estimated DBNs with 17 tasks at the first measurement time point and 9 tasks at the second time point from a sample size of 1,450. The following subsections illustrate inferences of various kinds that are supported by such a network.

#### Task inferences

A task was initially classified as being “at the level” if it supported an interpretation that students reaching that level would be able to solve or complete the task, whereas students at lower levels would be unlikely to be successful. To refine the classifications, the CPT of each task was examined. The results indicated that most of the tasks discriminated between the targeted level and the remaining levels. For example, Table 4 is the CPT for Task 7 at the first measurement as provided by Netica. The CPT shows clearly that students at level 2, level 3, and level 4 are likely to successfully solve Task 7, whereas students at level 1 are unlikely to successfully solve this task. The evidentiary value of this task is primarily in differentiating students between level 1 and the higher levels.

**TABLE 4:** Conditional probabilities for Task 7 at the first measurement (% Probability).

		Correct	Incorrect
LP_ Measurement1	Level 1	17.49	82.51
	Level 2	76.17	23.83
	Level 3	79.98	20.02
	Level 4	83.80	16.20

**FIGURE 7:**
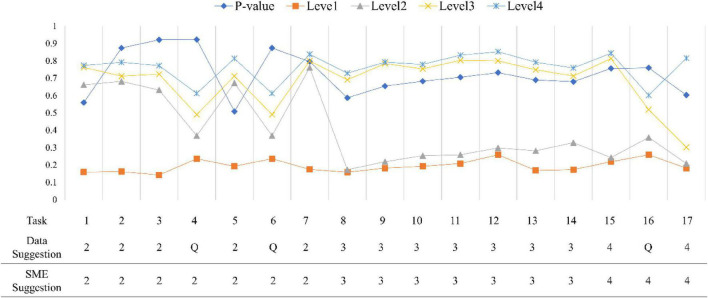
Conditional probabilities for all items at the first occasion. *P*-value indicates a proportion of correcting a task; SME is a subject matter expert group; Q indicates an ambiguous item.

**FIGURE 8:**
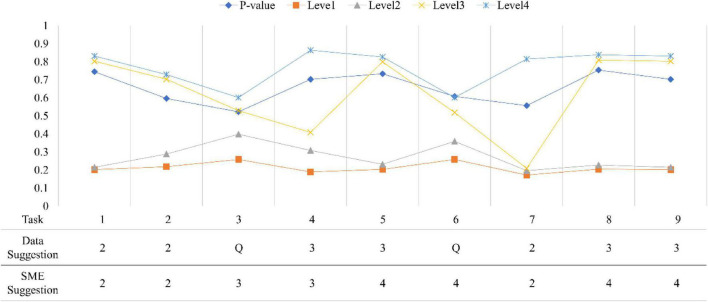
Conditional probabilities of each item at the second occasion. *P*-value indicates a proportion of correct responses to a task; SME is a subject matter expert group; Q indicates an ambiguous item.

Figures 7, 8 show all conditional probabilities of each task. Tasks 4, 6, and 16 at the first occasion while Tasks 3 and 6 at the second occasion were more ambiguous patterns in terms of their levels. For example, the conditional probabilities demonstrated a pattern where students at the lower level have little higher probability of completing the task correctly than students at the higher level.

Across all tasks, except Task 4, Task 6, and Task 16 at the first occasion and Task 3 and Task 6 at the second occasion exhibited clear and distinct patterns and were consistent with the experts’ expectations, meaning that they classified between levels as predicted by experts. Task 4, Task 6, and Task 16 at the first occasion and Task 3 and Task 6 at the second occasion were reported as ambiguous tasks for discriminating different levels in LP. In addition, Task 5, Task 8, and Task 9 at the second occasion (final exam) seemed to be mismatched with the experts’ expectations. That is, they were not located at the expected levels. Initial reviews of these results were passed on to content experts to provide feedback that would help them revise the tasks to more sharply target the concepts at their intended levels.

#### Inferences about individual students

**FIGURE 9:**
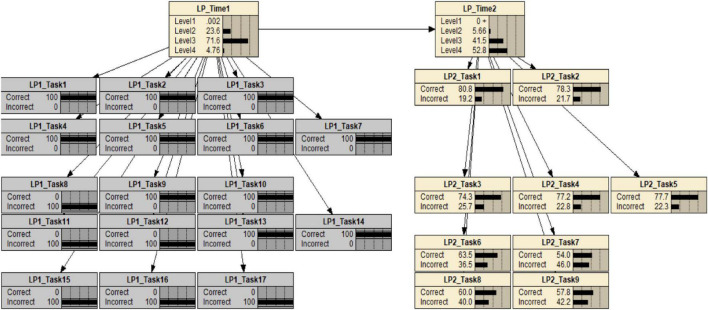
A DBN for a student who has completed 17 tasks at the first measurement.

Once the response pattern was observed, the CPTs in the DBN also provided information about student levels at the two measurement occasions. The information contained in a student response patterns is propagated through the network via Bayes’ theorem to yield posterior distributions of student levels on the LP. The posterior distribution provides the probabilities that a student has reached a specified level. On this basis, it can be inferred that the student is likely to have reached any given level. For instance, Figure 9 shows the DBN for a student who has completed 17 tasks at the first measurement.

The student has the response pattern of [1,1,1,1,1,1,1,0,1,1,0,0,1,1,0,0,0] at the first measurement. On the basis of this evidence, the posterior distributions for the student’s LP1 and LP2 indicate that the student has a probability of being at levels 1 to 4 of 0.002, 0.179, 0.807, and 0.146, respectively at the first measurement and a probability of being at levels 1 to 4 of 0.000, 0.048, 0.442, and 0.510, respectively, at the second measurement. On this basis, it may be inferred that the student is more likely to be level 3 at the first measurement and is more likely to be at level 3 or 4 at the second measurement.

The information in this network, used periodically during a student’s course in study, holds diagnostic utility for checking students’ progress along the curriculum. In particular predictions of low levels of future performance suggests a need for additional practice and review at the current LP level before proceeding to the successive levels. Finer-grained diagnostic information within a given level, for example in a unit of study focusing on selected concepts and skills at level.

#### Transition probabilities

In addition to the inference about a student’s level change over time, the DBN offers the probabilities of the transition between two measurements through the transition probability table. Table 5 is the transition probability table, which shows the probabilities of students having reached each level at the second measurement given their levels at the first measurement. For instance, a student has a 0.197 probability of being at level 2 at the second measurement point from being at level 1 at the first measurement point. For the backward transition movements, zero probabilities were estimated. That was because not only a constraint of no-backward movements was set using the prior information, but also the data were consistent with this expectation. With this information, one can infer the proportions of students that stay at the same level and move to the different levels between two consecutive measurements.

**TABLE 5:** Transition probability table (% Probability).

		LP_Measurement2			
		Level 1	Level 2	Level 3	Level 4
LP_ Measurement1	Level 1	19.3	19.7	23.8	36.2
	Level 2	0	24.0	29.6	43.4
	Level 3	0	0	48.2	51.8
	Level 4	0	0	0	100

#### Communicating with content experts

The results based on empirical data analysis can serve to aid the interpretation of the development of KSAs that constitute the LP and accompanying tasks to support inferences about students. In most cases, the results for tasks were consistent with the expert-based expectation (Table 6). For other tasks, the results were more ambiguous or suggest an alternative interpretation to that of the experts. The results of the data analysis may be taken back to the content experts for consultation and possible refinements in terms of the definition of the LP, the tasks that assess aspects of the LP, and the utility of revised tasks or additional tasks for modeling students’ progress.

**TABLE 6:** Communicating with content experts.

Task	Expectation	Data analysis	*P*-value	Experts comments	Level final decision
LP1_Task4	Level 2	Ambiguous	0.92	Knowledge Concept is in Level 2, but the distractors seem to be confusing.	Keep
LP1_Task6	Level 2	Ambiguous	0.87	Knowledge Concept is in Level 2, but the distractors seem to be confusing.	Keep
LP1_Task 16	Level 4	Ambiguous	0.76	Knowledge Concept is in Level 4, but cognitively simple question	Keep
LP2_Task3	Level 3	Ambiguous	0.82	Knowledge Concept is in Level 4, but cognitively simple question	Keep
LP2_Task5	Level 4	Level 3	0.73	Refined as level 3	Refined
LP2_Task6	Level 2	Ambiguous	0.87	Knowledge Concept is in Level 2, but the distractors seem to be confusing.	Keep
LP2_Task8	Level 4	Level 3	0.75	Knowledge Concept is in Level 4, but cognitively simple question	Keep
LP2_Task9	Level 4	Level 3	0.70	Knowledge Concept is in Level 4, but cognitively simple question	Keep

In the second measurement occasion, among the three tasks that were not consistent with the expert expectations, the content expert agreed that the level of Task 5 needed to be refined. Task 5 was originally identified as being level 4 by the content expert, but the data analysis suggested that the task would be useful for classifying students between levels 1 and 2 as opposed to levels 3 and 4. The content expert commented that Task 8 and Task 9 fit perfectly into the levels that have been originally identified although the data analysis suggested different levels. However, he pointed out that Task 8 requires a simple cognitive recall process to complete the task, which might make them easier than other tasks at the same levels. In other words, although the tasks measure a higher level of KSAs in terms of content, they required a lower level of cognitive ability to solve the tasks. This may be a possible reason that the data analysis suggested the lower level (level 3) than their expectation (level 4). The content expert felt strongly that task 9 should keep the same level originally identified. The task has a relatively higher proportion value of correcting the task (*P*-value = 0.7) than other tasks in the same level (level 4). Therefore, there may be other factors that influence the level of task difficulty such as difficult distractors and task format. Table 6 shows the summary of the agreement between expectation and data analysis. If only the *P*-value is considered to determine if a task is correctly located, it could not provide sufficient information. For instance, Task 1 at the first measurement seems to be incorrectly located based on the *P*-value (i.e., it has relatively low *P*-value: 0.56). However, the task performed very well for classifying the students between at level 1 and level 2, 3, and 4.

## Conclusion

### Contributions

Formative assessments are increasingly of interest in the field of education. A formative assessment system provides information for teachers and students about whether specific learning goals are being reached, and what is needed to master a given concept (Bennett, 2011; McCallum and Milner, 2020). For this purpose, an assessment must produce evidence for revealing student levels and their change over time (Black and Wiliam, 1998; Bennett, 2011). While substantive, psychological, instructional, and task developmental aspects of formative assessment have been considered, few assessment design frameworks have been proposed to link the theory embodied in a learning progression, tasks that provide evidence about a student’s level on that progression, and psychometric models that can link them. More specifically, few statistical analytic models have been studied to characterize the relationship between student performance and levels on learning progressions under a formative assessment system. In this study, we review a coherent assessment design framework for modeling learning progression under a formative assessment system using an ECD framework. Then we describe how DBNs can be used to addresses the question of how a learner’s current, past, and future levels in learning progressions are inferred under a formative assessment. Finally, we conduct an application study of DBNs using real data from the domain of beginning computer network engineering drawn from an online formative assessment in the CNA. Consequently, this study describes a design framework and learning analytics method for measuring students’ advancement along learning progression in a formative assessment system.

Dynamic Bayesian Networks are a useful statistical modeling method that can support inferences about level change over time when task design and theory provide not only a theoretical framework for creating and modeling observable evidence, but also information about the nature and structure of expected change. DBNs provide real-time updating of estimates for student levels during instruction, so that they offer beneficial information to students, instructors, and curriculum developers for enhancing student learning (Almond, 2007; Arieli-Attali et al., 2019; Attali and Arieli-Attali, 2019).

In addition, DBNs can serve as a psychometric model in research related to learning progressions. LPs inform the state of a student with respect to their level of understanding of a given concept and diagnostic information regarding the strengths and weaknesses of a student’s understanding along a curricular strand. However, challenges have emerged in LP research, including (1) designing a coherent assessment system, (2) inferring student learning progression levels based on the responses to assessment tasks, and (3) interpreting the difference between expected and observed students’ progress mapped to the conceptually defined learning progression. An ECD Framework can be a useful tool for modeling learning progressions by linking among the theory embodied in a progression, tasks that provide evidence about a student’s level on that progression, and psychometric models that can characterize the relationship between student performance and levels on learning progressions. Bayesian Networks can work as a psychometric model that can provide real-time updating of estimates for a student learning progression.

Furthermore, BNs can help lead to a valid task design. BNs confirm the levels and progressions by comparing the results from data analysis, allowing task designers to specify the levels of KSAs at which they are aiming assessment tasks. This helps make task design more principled, better connected with targeted inferences, and ultimately more valid. Lastly, BNs also help connect curriculum to assessment. For example, curriculum designers can take information from a BN structure and make decisions about which content areas are more important to emphasize so that students will have a greater probability of mastering future KSAs (West et al., 2010, 2012).

### Limitations and future study

Although BNs provide a promising means for modeling LPs under an assessment system with formative and summative components, there are some issues that need to be considered in the future study. First, since BNs are a flexible statistical framework, there are many decisions that must be made when designing a BN. The structure of the relation among variables must be determined. The structure can be determined by communicating with content experts as well as by using statistical methods such as structural equation modeling. In addition, one may need information about the probability distributions of variables in the BN as prior information. While the *values* of the variables of the probability distributions can be determined from data, the *structure* can be posited before data are collected (although it may be revised in light of subsequent data; see Levy, 2006). Therefore, it is important to keep in mind the purpose of the assessment in order to determine which relations are important to model. This process is not always straightforward and may require some iterative work before the model can be said to be good enough (Almond et al., 2015). Second, this study considered a first order Markov property in the transition probability. The designing of DBNs with more than two measurements with a higher order Markov property could reflect a more realistic educational setting.

Third, this study was initially designed with one LP variable corresponding to a domain. However, more than one LP reflecting more than one domain may be of interest to be modeled in future studies. For example, tasks can be reflected by more than one LP and students might have different learning patterns in terms of multi-LPs representing different domains. A student may require a higher level on one LP while additionally requiring a lower level on the other LP to complete a given task in the domain. In the case of multiple LPs, different learning paths along the multi-LPs can be modeled over time (Wilson, 2012).

Fourth, this study considered only the forward transition movement in the simulation studies because the forward transition movement is the most appropriate structure for representing student movements along an LP. This is a strong constraint but a typical hypothesis and appropriate for a preliminary study of DBNs for modeling LPs. However, there can be different types of transition structures, such as selected backward transition movements and all-transition movement. It is left to future studies to investigate the selection of the best fit transition movement structure for a particular content area, student population, and instructional program.

Lastly, the data example used to illustrate the DBM used only discrete-coded responses of conditionally independent tasks. The same LPs can be used to describe targeted KSAs, task demands, and student performance for more complex tasks such as the simulation-based troubleshooting and design tasks as are employed in CNA in the Packet Tracer environment (Williamson et al., 2004).

## Data availability statement

The raw data supporting the conclusions of this article will be made available by the authors, without undue reservation.

## Ethics statement

Ethical review and approval was not required for the study on human participants in accordance with the local legislation and institutional requirements. Written informed consent for participation was not required for this study in accordance with the national legislation and the institutional requirements.

## Author contributions

YC designed, analyzed, and wrote the manuscript. RM supervised the overall study process and direction. Both authors contributed to and approved the final version of the manuscript.

## Conflict of interest

The authors declare that the research was conducted in the absence of any commercial or financial relationships that could be construed as a potential conflict of interest.

## Publisher’s note

All claims expressed in this article are solely those of the authors and do not necessarily represent those of their affiliated organizations, or those of the publisher, the editors and the reviewers. Any product that may be evaluated in this article, or claim that may be made by its manufacturer, is not guaranteed or endorsed by the publisher.

## Footnotes

1 A DBN can be extended to an influence diagram to support instruction by additionally incorporating covariates as parents of the transitions, such as instructional treatment options, and decision nodes for selecting treatments (Almond, 2007; Arieli-Attali et al., 2019).
